# Production of ergothioneine by *Methylobacterium* species

**DOI:** 10.3389/fmicb.2015.01185

**Published:** 2015-10-27

**Authors:** Kabir M. Alamgir, Sachiko Masuda, Yoshiko Fujitani, Fumio Fukuda, Akio Tani

**Affiliations:** ^1^Group of Plant-Microbe Interactions, Institute of Plant Science and Resources, Okayama UniversityOkayama, Japan; ^2^Advanced Low Carbon Technology Research and Development Program, Japan Science and Technology AgencyTokyo, Japan; ^3^Laboratory of Pomology, Graduate School of Environmental and Life Science, Okayama UniversityOkayama, Japan

**Keywords:** ergothioneine, methylotroph, methanol, *Methylobacterium* species, glutathione, reactive oxygen species, antioxidant

## Abstract

Metabolomic analysis revealed that *Methylobacterium* cells accumulate a large amount of ergothioneine (EGT), which is a sulfur-containing, non-proteinogenic, antioxidative amino acid derived from histidine. EGT biosynthesis and its role in methylotrophy and physiology for plant surface-symbiotic *Methylobacterium* species were investigated in this study. Almost all *Methylobacterium* type strains can synthesize EGT. We selected one of the most productive strains (*M. aquaticum* strain 22A isolated from a moss), and investigated the feasibility of fermentative EGT production through optimization of the culture condition. Methanol as a carbon source served as the best substrate for production. The productivity reached up to 1000 μg/100 ml culture (1200 μg/g wet weight cells, 6.3 mg/g dry weight) in 38 days. Next, we identified the genes (*egtBD*) responsible for EGT synthesis, and generated a deletion mutant defective in EGT production. Compared to the wild type, the mutant showed better growth on methanol and on the plant surface as well as severe susceptibility to heat treatment and irradiation of ultraviolet (UV) and sunlight. These results suggested that EGT is not involved in methylotrophy, but is involved in their phyllospheric lifestyle fitness of the genus in natural conditions.

## Introduction

*Methylobacterium* species are facultative methylotrophic bacteria that can use both methanol and multi-carbon substances. They are ubiquitous in nature, and metagenomic studies on some plant species (Knief et al., 2011) and rice (Delmotte et al., 2009) showed their predomination on plant surfaces (the phyllosphere). This is believed to occur because plants emit methanol as a by-product of pectin demethylation (Fall and Benson, 1996; Jourand et al., 2005). Since global leaf area is estimated to be ca. 6.4 × 10^8^ km^2^ (Morris and Kinkel, 2002) and global emission of plant methanol is estimated to be 10^14^ grams per year (Guenther et al., 1995), the interaction between plants and *Methylobacterium* species is of importance to consider plant health, agriculture, and the global cycle of one-carbon compounds as well. In addition to the abundant and extensive study on their methylotrophic metabolism (Vuilleumier et al., 2009), their physiology in the phyllosphere has been gathering a great deal of attention (Gourion et al., 2006), since the phyllosphere is considered to be a harsh environment, in which microorganisms are exposed to UV, temperature shifts, fluctuations in water availability, and limited resources for growth (Vorholt, 2012). *Methylobacterium* species have been reported to be able to promote plant growth (Abanda-Nkpwatt et al., 2006; Tani et al., 2012) due to their ability to synthesize phytohormones (Ivanova et al., 2000; Koenig et al., 2002; Schauer and Kutschera, 2011) and 1-aminocyclopropane 1-carboxylate deaminase, which decreases the ethylene level in plants (Madhaiyan et al., 2006, 2007; Chinnadurai et al., 2009).

The microbial synthesis of low-value products as well as fine chemical compounds using methylotrophs has also been documented. Methanol is a cheap, non-food feedstock that is easily generated from diverse renewable sources such as biogas and synthesis gas. These gases can be derived from methane, which is also abundant and inexpensive. Thus, methanol is a more attractive and advantageous feedstock than sugars and their polymers. In addition, developments in fermentation technology (Bourque et al., 1995; Bélanger et al., 2004) as well as the accumulated knowledge on metabolic pathways (Šmejkalová et al., 2010; Chistoserdova, 2011) have been rendering methylotrophic bacteria as attractive catalysts to synthesize fine chemicals. The production of amino acids and polyhydroxyalkanoates as well as high biomass yield from methanol has been reviewed by Schrader et al. (2009).

In our previous report, we isolated *M. aquaticum* strain 22A from a hydroponic culture sample of a moss, *Racomitrium japonicum* (Tani et al., 2012). We have been using the strain as a model for *Methylobacterium*-plant interaction with respect to plant-growth promotion. We found that strain 22A cells highly accumulate ergothioneine (EGT) through metabolome analysis in this study. EGT is a sulfur-containing, non-proteinogenic amino acid derived from histidine. It was first discovered in ergot fungi and its structure was determined in 1911 (Mann and Leone, 1953). The compound is believed to be synthesized in few organisms, notably actinobacteria, cyanobacteria, and certain fungi (Fahey, 2001; Pfeiffer et al., 2011). The genes for EGT synthesis were first described for *Mycobacterium* species (Seebeck, 2010). The clustered *egtABCDE* genes were shown to encode proteins that convert histidine to EGT. EgtD is a methyltransferase that converts histidine to hercynine. EgtB, an FGE (formylglycine generating enzyme)-like protein, conjugates γ-glutamylcysteine to hercynine to form γ-glutamylcysteinylhercynine. EgtC, a glutamine amidotransferase, releases glutamate from it to generate S-(β-amino-β-carboxyethyl)ergothioneine sulfoxide. EgtE, pyridoxal 5-phosphate dependent β-lyase, forms EGT. EgtA (γ-glutamylcysteine synthetase) supplies γ-glutamylcysteine for EgtB. Homologous genes for *egtB* and *egtD* were found in many eukaryotes and bacteria, including *Methylobacterium* species. The other genes have also been detected in many eukaryotes (Jones et al., 2014). Thus, EGT synthesis in *Methylobacterium* species has been predicted, but its productivity and its role in methylotrophy and physiology have not hitherto been investigated.

EGT has been reported to be an important component of cells because of its antioxidant properties (Cheah and Halliwell, 2012). In humans, EGT has been shown to accumulate in various cells and tissues at high concentrations (100 μM—2 mM), although human cells do not produce EGT (Cheah and Halliwell, 2012). EGT is concentrated in mammalian mitochondria, suggesting a functional role in protecting it from mitochondrial superoxide (Paul and Snyder, 2010). EGT is known to absorb ultraviolet (UV) light, which may account for the ability to block UV damage (Bazela et al., 2014). Importantly, in mammalian cells, the EGT transporter ETT/OCTN1 was identified (Gründemann et al., 2005; Grigat et al., 2007), suggesting that EGT is preferentially acquired by the cells. Certain species of mushrooms are distinguished sources of EGT, ranging from 0.4 to 2.0 mg/g (Ito et al., 2011) or 0.08 to 3.78 mg/g (Dubost et al., 2006) in dry weight. In *Mycobacterium smegmatis*, intracellular and extracellular EGT of 4.1 and 17 pg/10^5^ colony-forming units (CFUs), respectively, was reported (Sao Emani et al., 2013). An overproduction system from the fission yeast *Schizosaccharomyces pombe* was constructed, resulting in 5000-fold higher productivity of 1600 μM in intracellular concentration (Pluskal et al., 2014). In cyanobacteria, 0.8 mg/g dry mass was reported (Pfeiffer et al., 2011).

In this study, we revealed the abundant EGT accumulation in *Methylobacterium* species for the first time and investigated the feasibility of fermentative EGT production from methanol using *M. aquaticum* strain 22A. Furthermore, we report the phenotype of a mutant deficient for EGT production, and suggest its important role in the phyllospheric lifestyle of the genus.

## Materials and methods

### Microbial strain and culture condition

Mineral medium (MM) composition is presented in Table S1. Different concentrations of methanol, succinate, glucose, or ethanol were used as carbon sources. R2A, Luria-Bertani (LB), and Middlebrook 7H9 (a mineral medium for *Mycobacterium* species containing sodium citrate and glutamic acid as carbon sources, on which *Methylobacterium* species grow well when methanol is supplemented) (Middlebrook and Cohn, 1958) were also tested. Kanamycin (25 μg/mL) was used when necessary. *Methylobacterium* species were cultivated at 28°C. *Escherichia coli* strains were grown in LB at 37°C.

A rifampicin-resistant spontaneous mutant of strain 22A (22A-rif) was obtained by streaking wild type on R2A agar containing 2 μg/ml rifampicin; its growth on methanol was confirmed to be normal. The frequency of spontaneous mutation was estimated to be 10^−7^. mTn5*gusA-pgfp22* (Xi et al., 1999) was used to derive the GFP-expressing kanamycin-resistant strain.

### Metabolome analysis with capillary electrophoresis time-of-flight mass spectrometry (CE-TOF/MS)

The cells of strain 22A (1.45 × 10^9^ cells) grown in liquid MM containing 0.5% methanol for 3 days were harvested, filter-trapped with a 0.4 μm pore membrane (millipore), and rinsed with water. The cells were transferred into 2 ml of methanol containing 5 μM methionine sulfone and camphor-10-sulfonic acid. The samples were sent to Human Metabolome Technologies Inc. (HMT, Tsuruoka, Japan). A total of 1.6 ml of chloroform and 640 μl of water were added to the sample. The sample was then vortexed and centrifuged at 2300 × g, at 4°C for 5 min. The water layer was taken and filtered by an Ultrafree-MC, 5 kDa (molecular weight) cut-off centrifugal filter device (HMT) to remove proteins. The filtrate was dried, dissolved in 25 μl of ultra-pure water, and then analyzed using CE-TOF/MS equipped with an Agilent 6210 TOF/MS (Agilent Technologies, Waldbronn, Germany). Cationic metabolites were analyzed with a fused silica capillary (50 μm inner diameter × 80 cm total length) with a commercial cation electrophoresis buffer (Solution H3301-1001, HMT) as the electrolyte. The sample was injected at a pressure of 50 mbar for 10 s (approximately 10 nl). The applied voltage was set at 27 kV. Electrospray ionization-mass spectrometry was conducted in the positive ion mode, and the capillary voltage was set at 4 kV. The spectrometer was scanned from 50 to 1000 m/z (mass-to-charge ratio). Other conditions were the same as those in the cation analysis described previously (Soga et al., 2003). In the same way, anionic metabolites were analyzed with a commercial anion electrophoresis buffer (Solution H3302-1021, HMT). The sample was injected at a pressure of 50 mbar for 25 s (approximately 25 nl). The applied voltage was set at 30 kV. Electrospray ionization-mass spectrometry was conducted in the negative ion mode, and the capillary voltage was set at 3.5 kV. The spectrometer was scanned from 50 to 1000 m/z. Other conditions were as in the anion analysis (Soga et al., 2003).

### EGT and glutathione quantification

The cells of strain 22A grown in liquid media were harvested by centrifugation (12,000 × g, 25°C, 10 min) and washed with 0.85% NaCl. The wet weight of the cells was recorded. Dry weight was obtained by complete drying of the cells at 100°C when necessary. The intracellular EGT was extracted by heating the cell suspension in water at 94°C for 10 min. The cell suspensions were vortexed at 1600 rpm for 30 min and centrifuged (14,000 × g, 25°C, 10 min) to remove cells. The supernatants were filtered (0.2 μm) and subjected to EGT quantification using a high performance liquid chromatography (HPLC). For extraction of glutathione, 600 μl of methanol was added to the 400 μl cell suspension. After mixing, 1 ml of chloroform was added. The sample was sonicated in a sonic bath for 1 min. A water layer of 1 ml was taken and subjected to HPLC analysis.

EGT was quantified by an HPLC equipped with an Asahipak NH2P-50 column (4.6 mm i.d. × 250 mm, Asahi Kasei Co.) attached with a guard column NH2P-50G, in the following conditions: injection volume, 20 μl; flow rate, 0.5 ml/min; detection, UV absorbance at 254 nm. Solvent A (50 mM sodium phosphate buffer containing 0.1% triethylamine, pH: 7.3) and solvent B (100 mM NaCl) were used to make a gradient: 0–7 min, 0% B, 7–8 min to 20% B, 8–13 min 20% B, and 13–15 min to 0% B. EGT is eluted at around 6.1 min.

Glutathione was quantified with the same instrument equipped with a reverse-phase μ-BONDAPAK C18 column (4.6 mm i.d. × 250 mm, Waters Co.) attached with a guard column (Inertsil WP300 C18 GL Science). Solvent A (0.1% trifluoroacetic acid) and solvent B (100% acetonitrile) were used in the isocratic solvent system of 30% solvent A at a constant flow rate of 0.75 ml/min. Glutathione was detected by UV absorbance at 210 nm, and was eluted at around 6.1 min (reduced form) and 6.6 min (oxidized form).

### Total amino acid analysis

Strain 22A culture grown in 100 ml MM containing 0.5% methanol for 7 days was separated into two fractions (10 and 90 ml). The former was used for EGT quantification as described above. The latter was used for total amino acid extraction. Cells were collected by centrifugation, and the methanol extract (2 ml) of the cells was dried in vacuo and dissolved in water (5 ml). The sample was applied to Amberlite CR1310NA (4 ml resin, pre-equilibrated with successive 1 M NaOH, water, 1 M HCl, and water). The resin was rinsed with water (50 ml), and amino acids were eluted by 2 M ammonia (40 ml). The eluent was dried in vacuo and dissolved in 5 ml water; 40 μl of the sample was analyzed using an amino acid analyzer (Hitachi L-8500B). Authentic EGT could not be quantified since the analysis depends on the ninhydrin reaction in the amino group of amino acids (the amino group is trimethylated in EGT).

### Generation of EGT mutant and complemented strains

Using the amino acid sequences of the *egt* genes of *Mycobacterium* species (Seebeck, 2010), *egt* gene homologs in the strain 22A genome (Tani et al., 2015) were found by basic local alignment search tool (BLAST) analysis. While *egtA, C*, and *E* are separately encoded in different loci, *egtB* and *egtD* are encoded in tandem in the strain 22A genome and they were subjected to deletion mutagenesis. Each kilobase of the upstream and downstream flanking regions of the *egtBD* genes was amplified using primers (5′ flanking sequence amplification; *egtB*_left_fw, tcgagctcggtacccatagagcaggctacgctgga and *egtB*_left_rv, catcggatcttccctcatgcg: 3′ flanking sequence amplification; *egtD*_right_fw, cgcatgagggaagatccgatcgtccaccatccggcggcactga and *egtD*_right_rv, ctctagaggatccccggtcgagctccatctccag). The fragments were tandemly cloned into the Sma I site in pK18mobSacB (Schäfer et al., 1994), using the In-Fusion cloning kit (Takara Bio Co.), yielding pK18mob-egtBD. Introduction of the plasmid into strain 22A was done via conjugation using *E. coli* S17-1. The kanamycin-resistant single-crossover mutant was streaked on R2A medium containing 10% sucrose to deliver double-crossover mutants (Δ*egt*), which were examined by diagnostic polymerase chain reaction (PCR). Δ*egt*(mTn5*gusA-pgfp22*) was generated by introducing mTn5*gusA-pgfp*22 into Δ*egt*, and its growth on methanol was confirmed to be normal.

For complementation of the mutation, the PCR fragment amplified from the wild-type genome with *egtB*_left_fw and *egtD*_right_rv, which contains functional *egtBD*, was cloned into pK18mobSacB. The resultant plasmid was introduced into Δ*egt*. Kanamycin-resistant colonies were selected for PCR diagnosis. The complemented strain was designated as Δ*egtComp*. The same plasmid was also introduced into the wild type, and the kanamycin-resistant strain was designated as *WTegtDup*.

### Phenotypic assays for Δ*egt*

Strain 22A wild type and Δ*egt* were grown in MM containing 0.5% methanol to the exponential growth phase. The cells were harvested by centrifugation and washed with fresh media to make a cell suspension of OD600 = 0.5. The cell suspension was used for the following assays, as described previously (Iguchi et al., 2013) with some modifications. (1) *Heat shock resistance assay*. Cells were spread on R2A agar plates for CFU determination after incubation of the cell suspension (100 μl) at 46°C for 5, 10, 15, and 20 min. (2) *UV resistance assay*. Cell suspensions (100 μl) were exposed to 253 nm UV light (GL-15, Toshiba Co.) for 2 min (distance 20 cm, equivalent to 800 μW/cm^2^), and then diluted and spread on R2A agar plates for CFU determination. (3) Sunlight resistance assay. Methanol-grown cells of ca. 200 CFU were spread on solid MM containing 0.5% methanol prepared in a glass dish. The plates were covered with a glass lid or wrapped with cellophane (thickness, 0.03 mm). The plates were placed on ice and exposed to sunlight at midday (done at IPSR Okayama University on Aug 18, 2015; 12:00 to 15:00; weather, sunny; air temperature, 33°C; photon flux, 1500 μmol s^−1^ m^−2^, measured with a quantum sensor, UIZ-PAR-LR, Uizin Co. Japan). After 0 and 3 h of exposure the plates were transferred into an incubator at 28°C in dark. The temperature of the plates did not exceed 26°C during the exposure. The colonies formed on the plates were counted after 7 days cultivation. (4) *Disk diffusion assay*. A cell suspension in 0.75% agar was overlaid on solid MM containing 0.5% methanol and 2% agar. Aliquots (5 μl) of 1 M H_2_O_2_, 2% methylglyoxal, or 1 M diamide were deposited on filter disks placed at the center of each plate. The diameter of the growth inhibition zone was measured. Student's *t*-test was used to evaluate the statistical significance of the differences in the bacterial counts.

### Growth of Δ*egt* on methanol

Strain 22A and Δ*egt* were cultured in 96-well plates containing 200 μl of MM containing methanol. The plates were incubated at 28°C without shaking. The cell growth (OD600) was measured using a microplate reader (Powerscan HT, DS Pharma Biomedical).

### In planta competition experiment

Sterile seeds of *Arabidopsis thaliana* Col-0 were placed on 1/2 Murashige-Skoog (1/2MS) agar medium containing 3% sucrose, 0.5% (v/v) MS(5) vitamin, and 0.8% agar (Ina Food industry, Co.), pH 5.6, prepared in plastic petri dishes. The rifampicin-resistant strain 22A-rif and kanamycin-resistant Δ*egt*(mTn5*gusA-pgfp22*) were inoculated onto the seeds. In the case of single inoculation, the inoculated cell suspension was 5 μl per seed, containing ca. 20,000 CFUs. For the competitive condition, a mixture of 2.5 μl of each suspension was inoculated. The plants were grown at 23°C under 16-/8-h light/dark conditions for 27 days. The shoots were excised and put in 500 μl water, and homogenized with pestles. The resultant suspension was spread onto selective R2A media containing kanamycin for Δ*erg* (mTn5*gusA-pgfp22*) and rifampicin for 22A-rif, for CFU determination.

## Results and discussion

### Metabolome analysis

The methanolic extract of methanol-grown strain 22A cells was subjected to metabolome analysis with CE-TOF/MS. We found that among the major metabolites like serine, glutamine, lysine, arginine, and glycine, EGT was detected as a highly accumulated metabolite (Table S2). However, since the compound was not included in the HMT quantifiable compound library, its quantity was not determined. We did another trial but it was unsuccessful because the internal standard was not detected for unknown reasons. However, the EGT peak area that was identifiable by migration time and mass-to-charge ratio in the trial was the highest among all detected compounds, suggesting the high accumulation of EGT (data not shown).

### Extraction and quantification of EGT

EGT separation and quantification by HPLC was successfully established, as described in the Materials and Methods section. We used heat treatment (94°C for 10 min) to effectively extract EGT from the cells without EGT decomposition. Additional heating of the remaining heat-treated cells also gave EGT, the amount of which was as small as 8% compared to that obtained by the first heating. We also varied the temperature of extraction (40–100°C), and found that 60°C was sufficient to extract most EGT (100% EGT was extracted compared to 100°C), and 50°C was insufficient (only 4.2% was extracted, data not shown). We used 94°C to ensure complete extraction for the following experiments. Heating of the aqueous EGT solution at 95°C did not result in apparent decomposition as monitored with HPLC (data not shown). Extraction with methanol resulted in a lowered EGT peak and the appearance of more other peaks in the HPLC chromatogram, suggesting that EGT was unstable in methanol. This might be the reason for the high variance of EGT concentrations detected in the metabolome analysis, where we used methanol for the extraction. We also quantified extracellular EGT in a concentrated spent medium (1-week-old 100 ml MM containing 0.5% methanol, re-dissolved in 1 ml water after completely dried, done in triplicate), but EGT content in the samples was under the detection limit (1 μM, injection volume to HPLC was 20 μl). Thus, extracellular EGT concentration was lower than 0.01 μM, suggesting that EGT is not secreted from the cells.

The simple method of heat treatment eases EGT extraction from the cells and reduces the downstream processing cost for EGT production. Although EGT has been reported to be secreted in *Mycobacterium* (Sao Emani et al., 2013), it was not secreted in *Methylobacterium* in this study. The difference in the physiological role of extracellular and intracellular EGT in different microorganisms remains unclear.

### Abundant accumulation of EGT in strain 22A

In standard conditions (5 ml MM containing 0.5% methanol, 7 days, 28°C), cell yield of strain 22A was usually approximately 30 mg (wet weight), equivalent to 6 mg dry cell weight. The EGT yield was usually 10 μg (1.6 mg EGT/g dry cell weight). The cell volume of strain 22A was estimated to be 0.65 μm^3^ (cell dimension was 2.24 × 0.65 μm bacilli form); therefore, the intracellular concentration of EGT was estimated to be approximately 183 mM. This intracellular concentration is much greater than the 0.3 μM that was detected in fission yeast cells and the 1.6 mM in the *P3nmt1-egt1*^+^ strain overexpressing the EGT synthetic gene (Pluskal et al., 2014). EGT productivity in strain 22A in this condition was comparable to that in the most productive mushroom, *Pleurotus osrteatus* (oyster mushroom, 3.78 mg/g dry weight) (Woldegiorgis et al., 2014).

**Figure 1:**
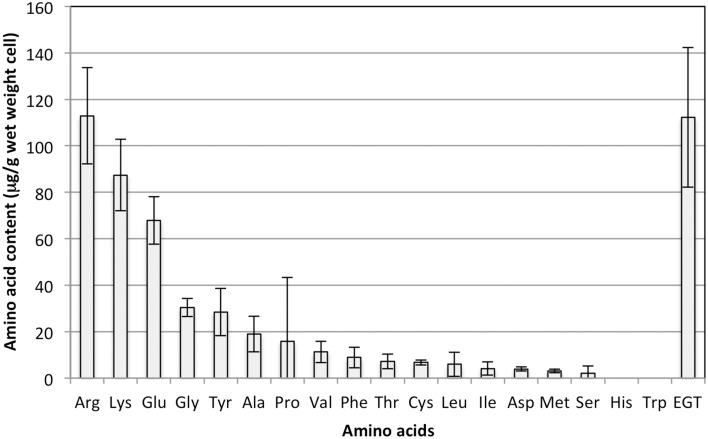
**Amino acid content in strain 22A cells grown on methanol (0.5%) for 7 days**. EGT was quantified by an HPLC and other amino acids were quantified using an amino acid analyzer. Glutamine and asparagine could not be analyzed by the analyzer. The data are presented as the mean ± standard deviation (SD) (*n* = 3).

As shown in Figure 1, the EGT content in the methanol-grown cells was one of the highest among amino acids quantifiable with the amino acid analyzer, again suggesting the unusually high accumulation of EGT.

### *M. aquaticum* strain 22A is one of the most EGT-productive strains among *methylobacterium* type strains

**Figure 2:**
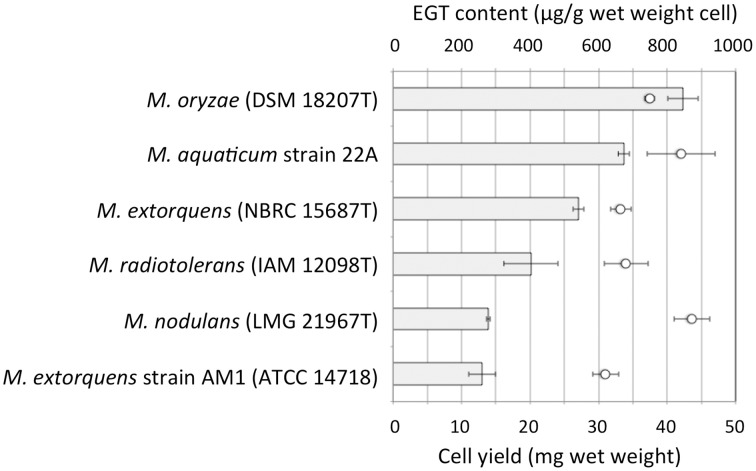
**EGT content in selected *Methylobacterium* type strains**. The strains were grown on 3% methanol in MM, and subjected to intracellular EGT quantification after 7 days of cultivation. The experiment was done in triplicate. The data are presented as the mean ± SD (*n* = 3). Bar, EGT content (μg/g wet weight cell), and circles, cell yield (mg wet weight).

We examined whether other *Methylobacterium* species also synthesize EGT. *Methylobacterium* type strains were grown on 0.5% methanol and intracellular EGT was quantified in the same condition. Through this preliminary screening, we found that most of the type strains synthesize EGT in a range of 0–100 μg/g wet weight cells (Figure S1). The productivity is widely distributed in *Methylobacterium* species and no bias was recognized in the relationship between their productivity and a phylogenetic tree based on their 16S rRNA gene sequences (data not shown). We selected five strains, four of whose genome information was available, and quantified EGT content with higher methanol concentration (3%) in triplicate. As shown in Figure 2, *M. oryzae* DSM18207 was the most productive and strain 22A was the second best. Since the genome information of *M. oryzae* was not available at that time (Kwak et al., 2014), we decided to use strain 22A for further analysis.

The genes for EGT synthesis have been found in many α-proteobacteria (Seebeck, 2010) and indeed many species of *Methylobacterium, Bradyrhizobium, Rhodopseudomonas*, etc. harbor the genes. There might be a more productive strain in the class. Since many of them are plant-symbionts, it is tempting to speculate that the EGT found in plants (Ey et al., 2007) might be derived from such symbiotic α-proteobacteria or that plants might also have a different biosynthetic pathway for EGT. Moreover, it has been reported that the EGT content in the orchid *Gastrodia elata* is correlated with the concentration of EGT in its symbiotic fungi, *Armillaria mellea* (Park et al., 2010).

### Optimization of EGT production

**Figure 3:**
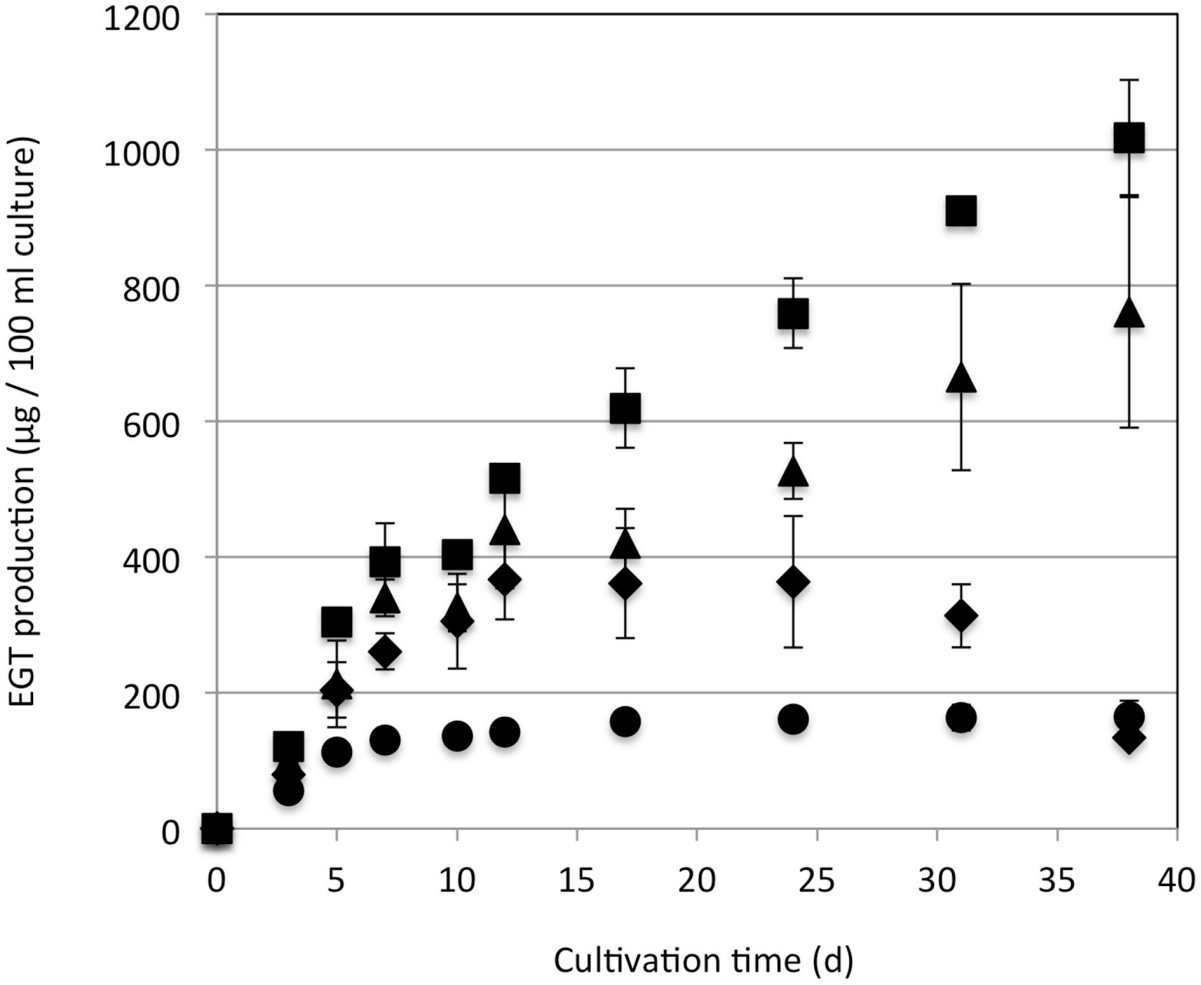
**EGT productivity of *Methylobacterium* sp. strain 22A**. The strain was grown on 0.5% (circles), 1% (diamonds), 2% (squares), and 3% (triangles) methanol in a 100 ml MM for 38 days. The experiment was done in triplicate. The data are presented as the mean ± SD (*n* = 3).

**Figure 4:**
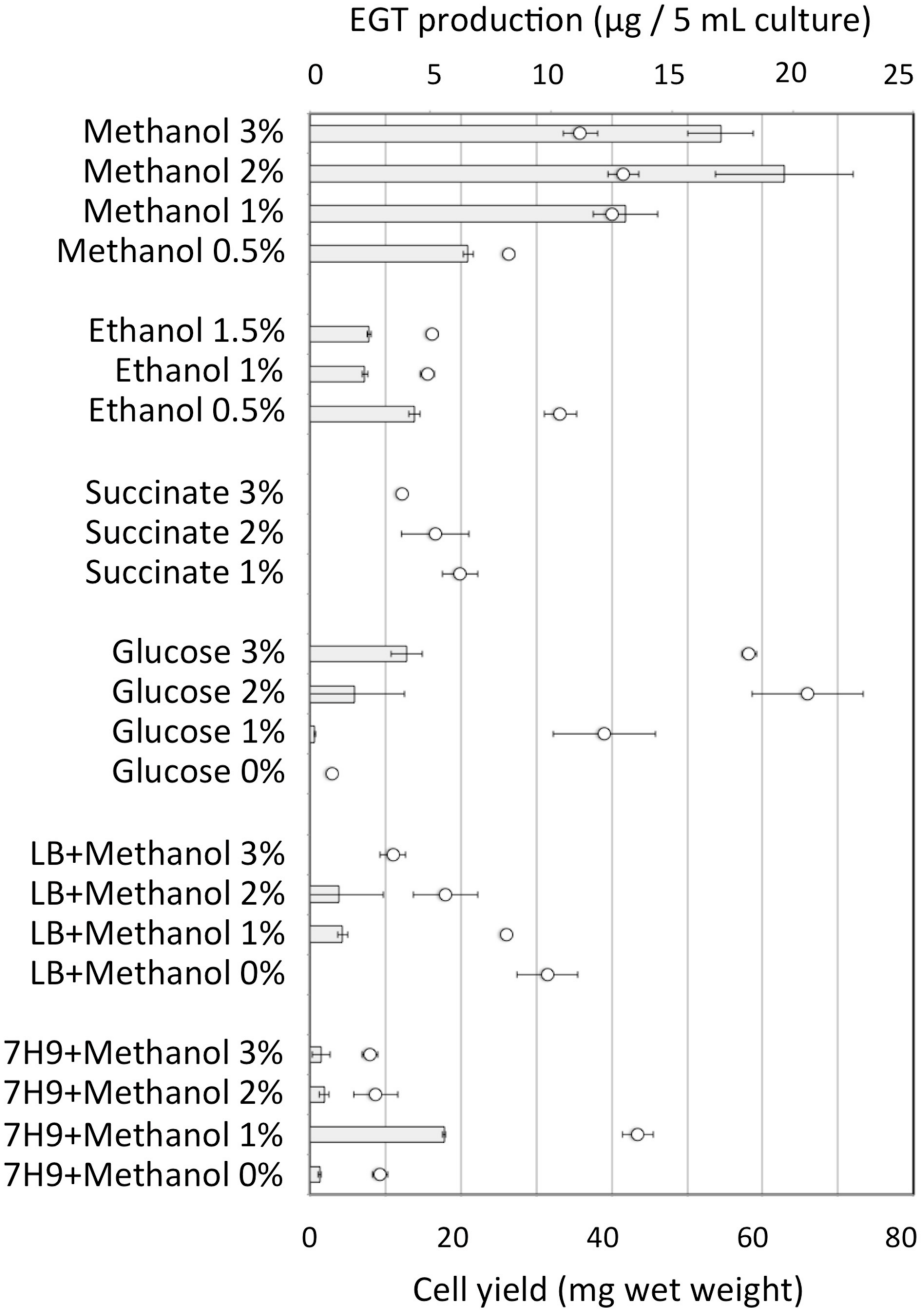
**EGT productivity of *Methylobacterium* sp. strain 22A grown on various carbon sources and media**. The experiment was done in triplicate using 5 ml media. The data are presented as the mean ± SD (*n* = 3). Bar, EGT production (μg/5 ml culture), and circles, cell yield (mg wet weight).

Strain 22A was cultured in MM containing 0.5% methanol (100 ml) containing different concentrations (0.5, 1, 2, and 3%) of methanol for 38 days and EGT productivity was monitored (Figure 3). Within a week, the cultures reached the stationary phase and OD600 increased only gradually afterwards (data not shown). Interestingly, the EGT amount in the culture did not increase after 7 days of cultivation on 0.5% methanol, but it increased when a higher concentration of methanol was used, whereas the OD600 did not increase. Strain 22A could grow in the presence of 3% methanol, but EGT productivity was best when 2% methanol was used. The EGT quantity in the MM containing 2% methanol reached 1000 μg/100 ml culture (1.2 mg/g wet weight, 6.3 mg/g dry weight) in 38 days, which overwhelms the productivity of *P. ostreatus*. EGT accumulated continuously until the late stationary phase, suggesting that EGT is a kind of secondary metabolite that is not involved directly in primary metabolism. Next, we tested other carbon sources, including ethanol, succinate, and glucose, and media of LB or Middlebrook 7H9 supplemented with methanol. EGT productivity was best when methanol was used (Figure 4). Succinate did not induce EGT production but glucose and ethanol did, suggesting that EGT production is not only induced by methanol. This result also suggested that EGT is not necessary for growth on methanol.

We tested other nitrogen sources at concentrations equivalent to 30 mM nitrogen, and found that ammonium nitrate, ammonium chloride, and ammonium sulfate gave comparable cell yield and EGT. (Figure S2). Then we changed the nitrogen source (ammonium chloride) concentration under 0.5% methanol, and found that 0.4–1.2 g/l was the best for production (Figure S3). Concentrations below 0.2 and above 2.0 g/l resulted in poor cell growth. Compared to carbon sources, nitrogen sources were less effective for EGT production.

Histidine and cysteine are the precursors in the EGT biosynthesis pathway (Seebeck, 2010). The supplementation of histidine, cysteine, and their combination did not enhance EGT productivity significantly (Figure S4). However, the combination at 10 mM doubled the productivity, but this concentration of amino acids is too high to be used as a supplement for large-scale EGT production. It has been shown that EgtB is dependent on iron (II) for its catalysis (Seebeck, 2010). Thus, we varied the FeSO_4_ concentration (0, 10, 17.3 [original concentration], 100, and 1000 μM) in MM containing 2% methanol. EGT production was comparable among 10–100 μM and decreased in 0 and 1000 μM FeSO_4_ (data not shown). *Methylobacterium* species usually possess two methanol dehydrogenases encoded by MxaF and XoxF; the former is calcium-dependent and the latter is lanthanide-dependent (Hibi et al., 2011; Nakagawa et al., 2012; Keltjens et al., 2014). Thus, the effect of lanthanide supplementation on EGT production was investigated under different methanol concentrations. The EGT production and growth were negatively impacted by a higher concentration of lanthanide (Figure S5), suggesting that lanthanide is not effective at enhancing production.

Thus, through the preliminary optimization of cultivation conditions, we found that 2% methanol with His and Cys supplementation at 10 mM was the best condition. Further optimization will be possible by testing other carbon sources and their mixtures, different temperatures, supplementation with other amino acids, and continuous cultivation with maintained pH and dissolved oxygen. Since EGT is considered to be a secondary metabolite, it may be necessary to mimic the condition of an aged culture to enhance the production, the factors of which are currently unknown.

### EGT genes found in *Methylobacterium* species

Genes homologous to *egtABCDE* in *Mycobacterium* (Seebeck, 2010; Jones et al., 2014) were found in strain 22A genome (Tani et al., 2015) by BLAST analysis, as well as in the complete genomes in other *Methylobacterium* species (Table S3). The overall shared identities based on amino acid sequences were 25–46% compared to those in *Mycobacterium* species. EGT biosynthesis genes were present as single copies in the strain 22A genome. *egtB* and *egtD* are found to be encoded tandemly in the largest plasmid (pMaq22A-1) in the strain 22A genome, whereas *egtA, C*, and *E* are separately encoded in distant loci in the chromosome (Figure S6). It is reported that the clustered *egtABCDE* genes are found only in actinomycetes, and that *egtB* and *egtD* are under strong selective pressure for genetic clustering (Jones et al., 2014). The cluster is also found in the genomes of *M. radiotolerans* JCM2831, *M. nodulans* ORS2060, *M. extorquens* AM1, and *Methylobacterium* sp. 4–46, but not in those of *M. oryzae* CBMB20 and *M. populi* Bj001. It is unknown whether their EGT productivity is dependent on the gene organization. However, since EgtD and EgtB catalyze the first two steps in EGT synthesis (Seebeck, 2010), the cluster may be important for efficient EGT synthesis. The conservation of five genes in *Methylobacterium* genomes supported their common ability to synthesize EGT in the genus.

### EGT is involved in resistance to heat shock, UV radiation, H_2_O_2_, and fitness for growth on plants

**Figure 5:**
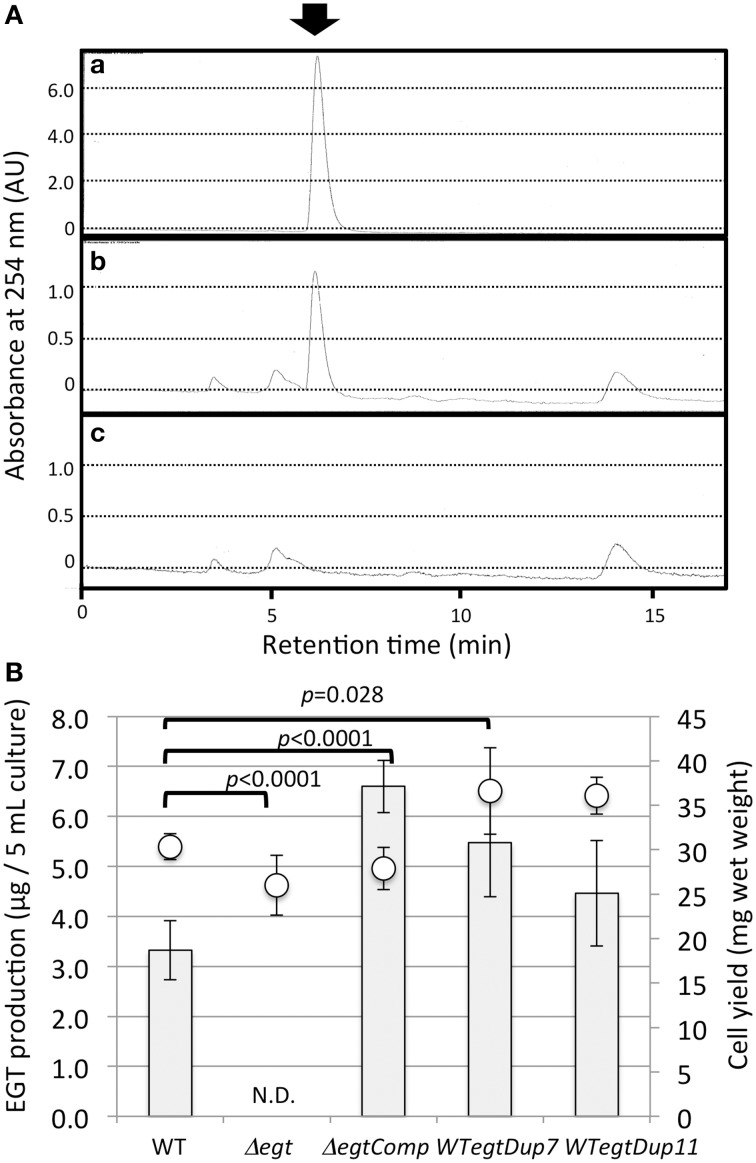
**(A)** HPLC chromatograms of (a) standard EGT (100 μM), (b) extract of strain 22A wild-type cells, and (c) extract of Δ*egt* cells. The arrow indicates the EGT peak detected at 6.2 min. The HPLC analysis condition is described in the text. **(B)** EGT productivity of Δ*egt*, Δ*egtComp*, and *WTegtDup cells*. Two independent transformants of *WTegtDup* isolates were tested. The cells were grown on 0.5% methanol in MM medium for 1 week and subjected to EGT quantification. Bar, EGT production (μg/5 ml culture), and circles, cell yield (mg wet weight). The data are presented as the mean ± SD (*n* = 5). *p*-values were generated by analysis of variance (ANOVA) using the Dunnett's multiple comparison test with the control (the wild type) for EGT production.

To clarify the function of EGT in *Methylobacterium*, we constructed an *egtBD*-deletion mutant (Δ*egt*) via homologous recombination. Furthermore, functional *egtBD* were introduced into Δ*egt* for complementation (Δ*egtComp*) and into the wild type as well for gene duplication (*WTegtDup*). EGT production in Δ*egt* was completely abolished, and Δ*egtComp* recovered EGT production with higher production than the wild type (Figure 5). Duplication of *egtBD* resulted in increased synthesis of EGT compared to the wild type. These results indicated that *egtBD* genes are indeed involved in EGT synthesis and that the synthetic pathway would be the same as the one for *Mycobacterium* species. In addition, it was possible to enhance the production by duplicating the genes.

The mutant Δ*egt* showed a slightly increased growth rate and cell yield on methanol (Figure S7), which was more evident with higher methanol concentration, indicating that EGT is not required for methylotrophic growth. When they were grown on methanol at elevated temperature (31°C and 34°C), slightly increased growth of Δ*egt* was still observed (data not shown). The reason for the better growth would be the biological cost for EGT production. Interestingly, the growth on glucose was not affected by the mutagenesis at all (data not shown), which was congruent with the low productivity of EGT on glucose (Figure 4).

**Figure 6:**
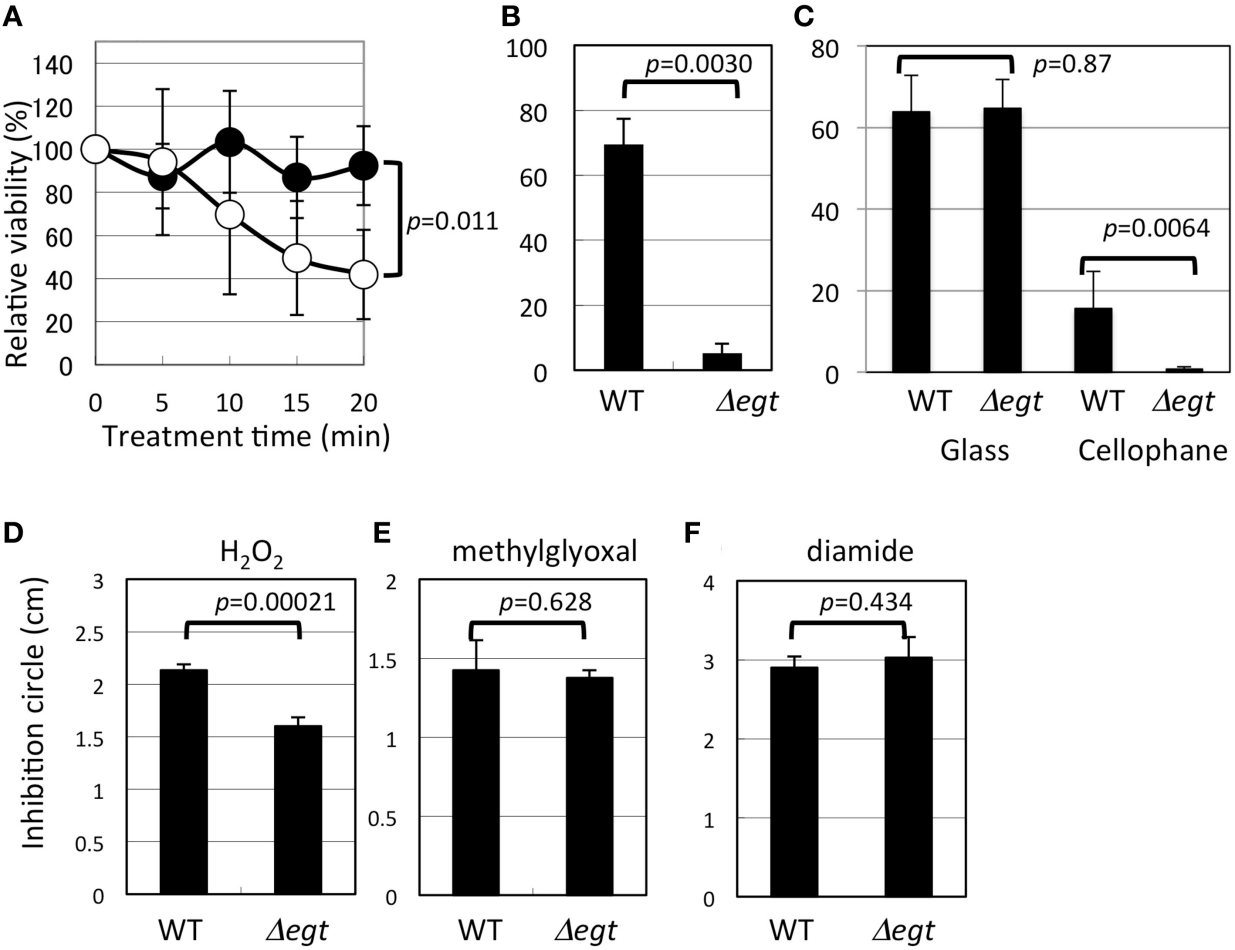
**Phenotypic characterization of Δ*egt* mutant. (A)** Heat shock assay at 46°C (closed symbols, wild type, and open symbols, Δ*egt*); **(B)** UV (253 nm) resistance assay; **(C)** sunlight-resistance assay; **(D–F)**, disk diffusion assay using hydrogen peroxide, methylglyoxal, and diamide, respectively. All experiments except **(C)** that was done in quintuplicate, were done in quadruplicate. The data are presented as the mean ± SD (*n* = 4). Experimental conditions are written in the text.

Δ*egt* was more sensitive to heat shock than the wild type (Figure 6A). In addition to heat shock, UV irradiation severely reduced the viability of Δ*egt* (Figure 6B). Exposure of 100 μM EGT solution to UV in the same condition resulted in a marked decrease in the EGT peak (35 and 87% decrease in 5 and 30 min, respectively) and a concomitant increased peak at 10.3 min (unknown compound) in the HPLC chromatogram, suggesting that EGT is degraded by UV (data not shown). Furthermore, when methanol-grown 22A cells were exposed to UV in the same condition for 5, 30, and 60 min, the intracellular EGT content decreased to 31, 0.5, and 0%, respectively (data not shown). These results indicated that EGT protects the cells for survival under UV. These results prompted us to see the effect of sunlight on the mutant survival. It is known that the strongest UV, UV-C (100–290 nm), does not reach to the ground due to the ozone layer. The UV lamp (254 nm) we used for the assay belongs to UV-C. Within the total UV reaching the ground, more than 95% is UV-A (320–400 nm), and less than 5% is UV-B (290–320 nm) that can cause DNA damage and kill bacteria. It is also known that glass is transparent to UV-A but not to UV-B (Duarte et al., 2009), and that cellophane is partly transparent to UV-B (Auras et al., 2004). We found that sunlight through the glass killed the wild type and the mutant at a comparable rate, and that sunlight through the cellophane killed Δ*egt* more efficiently than the wild type (Figure 6C). We also observed that the mutant formed colonies much slower than the wild type after the exposure (data not shown). These results strongly suggested that EGT protects the cells against sunlight, to which the *Methylobacterium* cells are exposed in the phyllosphere.

Interestingly, Δ*egt* showed higher resistance to H_2_O_2_ (Figure 6D), but no phenotype in sensitivity to other oxidative stress of methylglyoxal and diamide (Figures 6E,F). It is known that *Methylobacterium* species synthesize glutathione in addition to EGT, and that *Mycobacterium* species synthesize EGT and mycothiol as sulfur-containing antioxidants. In *Mycobacterium smegmatis*, mycothiol-deficient mutants showed elevated levels of EGT (Ta et al., 2011), whereas mycothiol level was unchanged in EGT mutant in *M. smegmatis* (Sao Emani et al., 2013). In contrast, *egtA* mutant in *Streptomyces coelicolor* A3(2), which still produces a decreased amount of EGT, produces five-fold more mycothiol compared to the wild type (Nakajima et al., 2015). Thus, in actinobacteria, a defect in one antioxidant may be compensated by an increased amount of another antioxidant. These facts led us to quantify glutathione in Δ*egt*. The glutathione content (reduced form) in Δ*egt* grown on methanol was 2.29 μg/5 ml culture, while that in the wild type was 2.05 μg/5 ml culture, showing a statistically insignificant increase (*n* = 3 each, *p* = 0.11, Student's *t*-test). The oxidized form was not detected. At this time, we cannot attribute the increased H_2_O_2_ resistance of the mutant to increased glutathione content. In the case of *egtA* mutant in *S. coelicolor* A3(2) (Nakajima et al., 2015), intracellular EGT content was severely reduced but the secreted EGT was not, and the mutant showed high susceptibility to H_2_O_2_. Thus, the resistance to H_2_O_2_ cannot be explained solely by intracellular EGT content for different bacteria, and it is possible that the strain 22A may contain other antioxidant molecules that compensate for the defect in EGT. A recent paper described the essential role of EGT and mycothiol in lincomycin A synthesis in *Streptomyces lincolnensis* (Zhao et al., 2015). Thus, it is also possible that *Methylobacterium* species utilize EGT for synthesis of other unknown secondary metabolites.

Taken together, these results indicate that EGT contributes to resistance to heat shock and UV stress. The antioxidant activity of EGT has been shown in many reports (Cheah and Halliwell, 2012). EGT reacts with the hydroxyl radical at a diffusion-controlled rate, but not readily with H_2_O_2_ and O${}_{2}^{-}$. Furthermore, EGT can repair the guanyl radical, which is a product of DNA ionization (Asmus et al., 1996). This could be the reason why Δ*egt* showed susceptibility to UV irradiation that elicits DNA damage.

Formaldehyde is the central metabolite in methylotrophy and it is not known whether EGT reacts with formaldehyde. Glutathione, on the other hand, is known to spontaneously react with formaldehyde to form S-hydroxymethylglutahione in *Paracoccus denitrificans* (Goenrich et al., 2002). In formaldehyde dissimilation in *Methylobacterium*, formaldehyde reacts with dephosphotetrahydromethanopterin (d_4_HMPT) either spontaneously or enzymatically by formaldehyde-activating enzyme (Fae). In assimilation, methylene tetrahydrofolate (H_4_F), as the C1 unit for the serine cycle, is produced either spontaneously by the condensation of formaldehyde and H_4_F or enzymatically through the H_4_MPT-dependent pathway (Ochsner et al., 2015). Thus, in *Methylobacterium*, glutathione is not involved in formaldehyde assimilation and dissimilation. However, recalling the case of *P. denitrificans*, we examined whether EGT reacts with formaldehyde. We mixed 1 M formaldehyde and 100 μM EGT, incubated it for 5–24 h at room temperature, and then examined the HPLC pattern. We did not observe any change in peak height or retention time, suggesting that EGT does not spontaneously react with formaldehyde *in vitro*. This result again indicates that EGT is not directly involved in methylotrophy.

**Figure 7:**
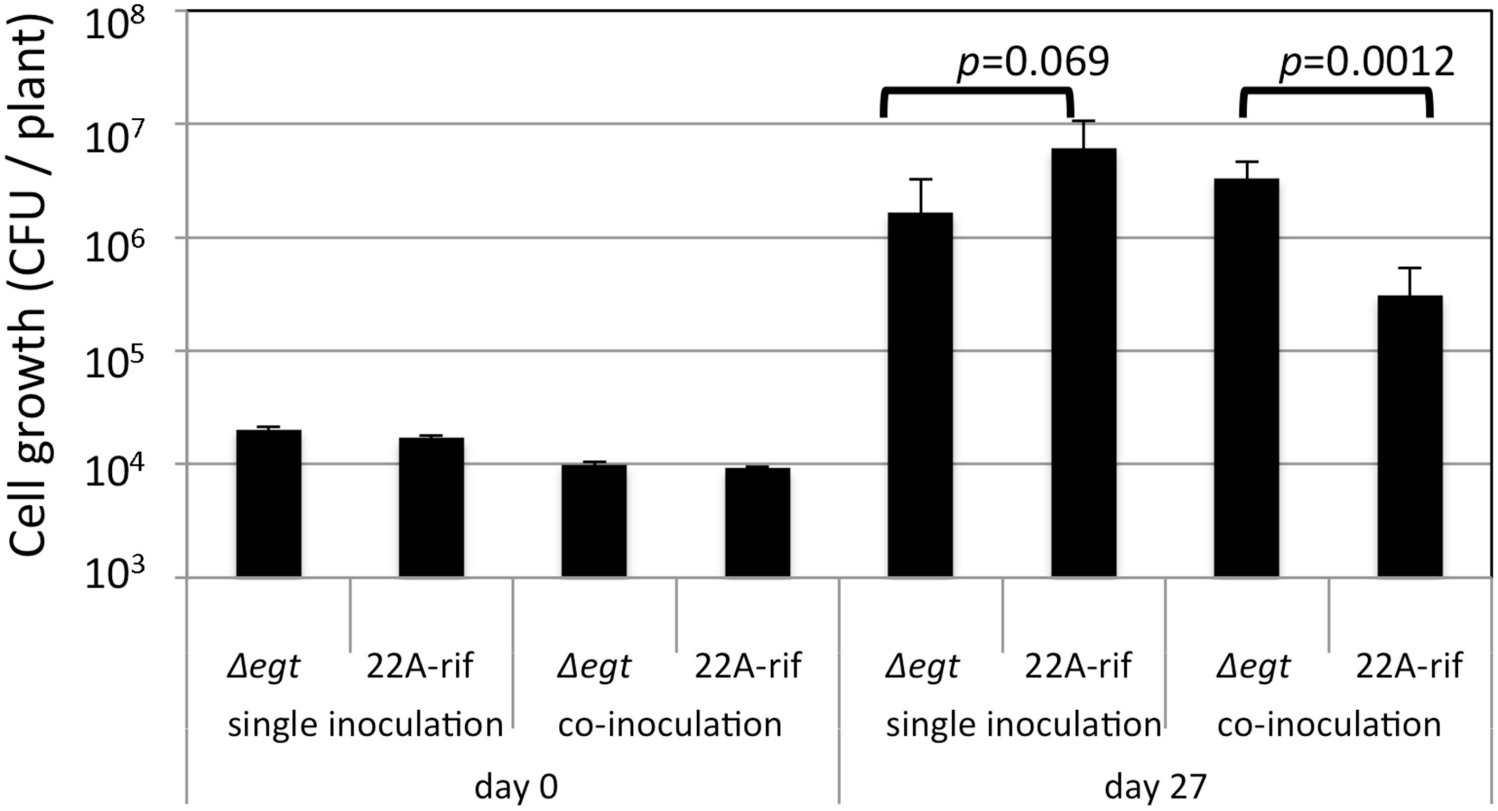
**Growth of strain 22A-rif and Δ*egt* inoculated onto *A. thaliana* seeds**. After 27 days of inoculation, the shoots were sampled and subjected to CFU determination using selective media. The data are presented as mean ± SD (*n* = 5).

Next, we examined the growth of Δ*egt* in the phyllosphere. The growth of Δ*egt* (mTn5*gusA-pgfp22*) was slightly decreased compared to 22A-rif when they were independently grown on *A. thaliana* leaves (Figure 7). When they were co-inoculated in competitive conditions, the lesser growth of 22A-rif was observed. As previously shown in Figure S7, the biological cost of EGT synthesis might be the reason for the lesser growth of the wild type. In this experimental condition, we used fluorescent lamps, which usually contain little UV. In addition, plastic petri dishes diminish UV. Thus, the experiment was performed in UV-free conditions.

### Exogenous supplementation of EGT is not effective in the growth of wild type and plant

Supplementation of EGT did not enhance the growth of the wild type on methanol, and was rather inhibitory (Figure S8A). The cause of this effect is unknown, but it is possible that EGT may inhibit some periplasmic enzymes, to which many of those involved in methanol oxidation belong. It is also unknown whether the cells incorporate EGT via transporters, since EGT is believed to be membrane-impermeable. We found proteins homologous to human OCTN1 (BAA23356.1) encoded in *Methylobacterium* genomes with less than 27% identity. This should be further examined to determine their involvement in EGT uptake.

The growth of *A. thaliana* on 1/2 MS agar was not affected by the exogenous supplementation of EGT at the tested concentrations (Figure S8B). Since EGT is exclusively accumulated in the cells and is not secreted extracellularly, exogenous EGT may not occur in *Methylobacterium*-plant interactions. Lysed bacterial cells may serve as an EGT source for plants, but there have been no reports on the existence of an active EGT transporter in plants. There are many OCTN1 homologs encoded in the genomes of a wide variety of plants, including cucumber, chickpea, wheat, barley, grapevine, potato, *Physcomitrella*, and so on, with less than 33% identity. It is currently unknown whether any of them is involved in EGT transport in plants. Since EGT is also found in plants, there must be an EGT transporter in plants; however, the biological role of EGT in plants remains uninvestigated.

In conclusion, we found that *Methylobacterium* species are able to synthesize abundant EGT. EGT is exclusively accumulated in the cells and is not secreted extracellularly. Using one of the most productive strains, we optimized the culture conditions mainly with respect to carbon and nitrogen sources and supplements. The production level overwhelms that of the most productive mushroom. High cell density cultivation is reported for *Methylobacterium* species, which can achieve as much as 100 g dry weight cells per liter (Bourque et al., 1995). These established cultivation techniques and the genetic manipulation we showed by gene duplication would be very useful in terms of improving production. Since pure EGT is expensive due to limited sources, we are interested in the further development of more efficient fermentative production using *Methylobacterium* species and methanol as a cheap feedstock. We identified EGT synthesis genes in strain 22A and generated an EGT synthesis mutant. EGT synthesis was found to be non-essential for methylotrophy. We also found that the mutant was susceptible to UV and heat shock. Importantly, the mutant is more susceptible to sunlight than the wild type. As inhabitants on the plant surface, resistance to UV is essential for their survival. Herein, we provided evidence of the involvement of EGT in the resistance. Since EGT was produced by many *Methylobacterium* type strains, EGT might be a ubiquitous UV-protectant for the genus.

Interestingly, EGT is a precursor for the synthesis of selenoneine, which has a selenoketone structure instead of thiocarbonyl in EGT (Yamashita et al., 2010). Selenoneine is reported to have three orders of magnitude stronger radical scavenging activity against 1-diphenyl-2-picrylhydrazyl than EGT (Yamashita and Yamashita, 2010). The synthesis of selenoneine is suggested to be dependent on selenocysteine (Pluskal et al., 2014). Further study is necessary to examine the possible potential of selenoneine synthesis and its role in *Methylobacterium* species. In addition, an EGT-degrading enzyme called ergothionase has been reported for *E. coli* (Wolff, 1962) and *Burkholderia* (Muramatsu et al., 2013). We found the homologous gene in the 22A genome, the mutant of which may be more productive.

## Author contributions

KA, SM, and AT designed the experiments. KA, SM, YF, FF, and AT performed the experiments. KA, SM, FF, and AT wrote the manuscript.

### Conflict of interest statement

The authors declare that this research was conducted in the absence of any commercial or financial relationships that could be construed as a potential conflict of interest. AT is listed as an inventor on a pending patent application (JP P2014-259232, filing date: December 22, 2014) on the use of Methylobacterium species for EGT production.
