# Supplementary material for: Production of ergothioneine by Methylobacterium species
Source: Front Microbiol. 2015 Oct 27;6:1185. doi: 10.3389/fmicb.2015.01185 (PMC4621440; doi:10.3389/fmicb.2015.01185)
Supplement: Supplementary file 4 [file DataSheet1.DOCX]

Supplementary data

**Production of ergothioneine by *Methylobacterium* species**

**Kabir Md Alamgir^1^, Sachiko Masuda^1,2^, Yoshiko Fujitani^1^, Fumio Fukuda^3^, and Akio Tani^1^***

^1^Group of Plant-Microbe Interactions, Institute of Plant Science and Resources, Okayama University, Okayama, Japan

^2^Advanced Low Carbon Technology Research and Development Program, Japan Science and Technology Agency, Tokyo, Japan

^3^Laboratory of Pomology, Graduate School of Environmental and Life Science, Okayama University, Okayama, Japan

**Correspondence**: Dr. Akio Tani, Group of Plant-Microbe Interactions, Institute of Plant Science and Resources, Okayama University, 2-20-1 Chuo, Kurashiki, Okayama 710-0046, Japan

atani@okayama-u.ac.jp


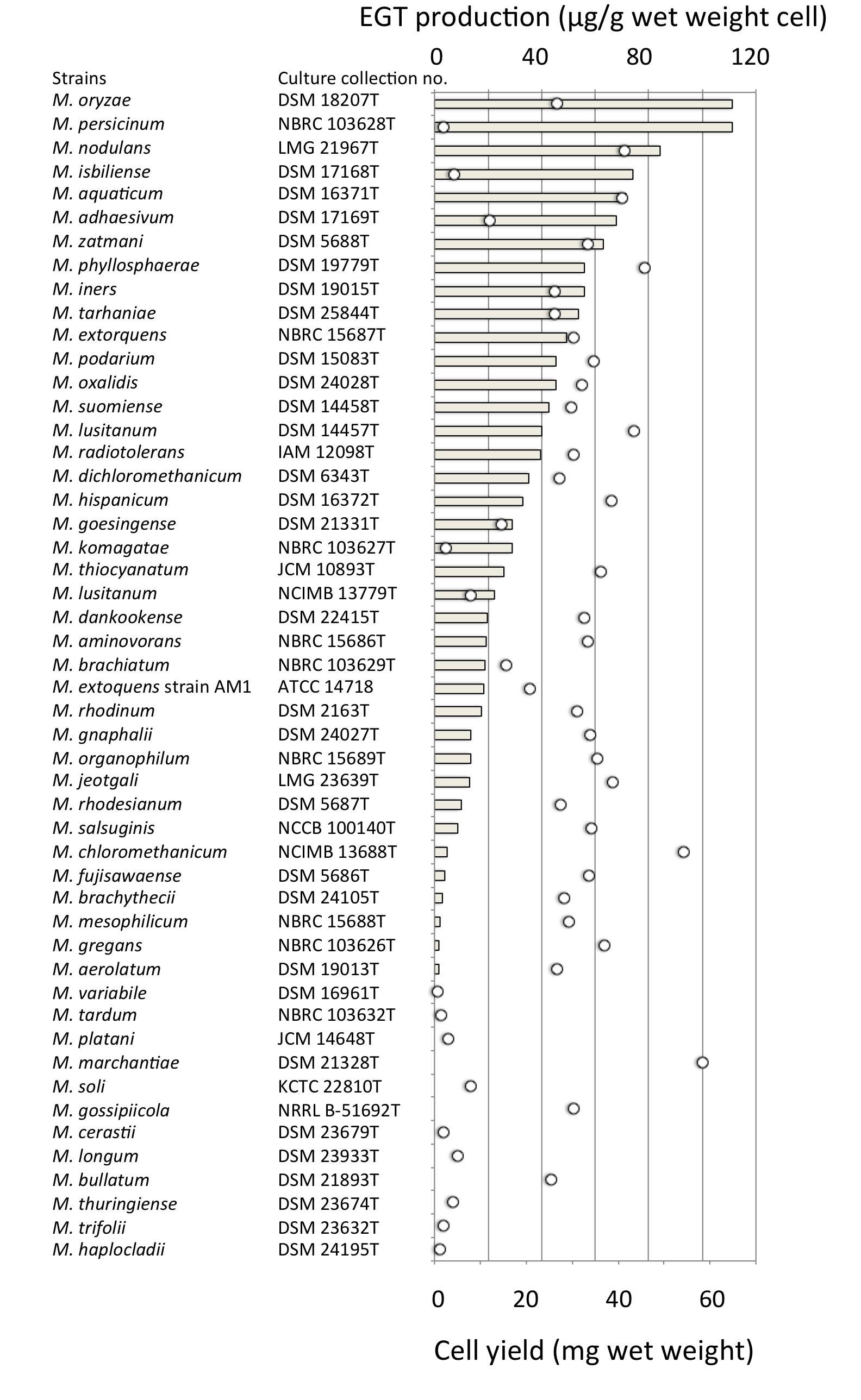


Figure S1. EGT content of *Methylobacterium* type strains. The type strains were grown on MM containing 0.5% methanol, and subjected to intracellular EGT quantification after 7 days of cultivation. The experiment was done without replicates. Bar, EGT production (µg/5 ml culture), and circles, cell yield (mg wet weight).


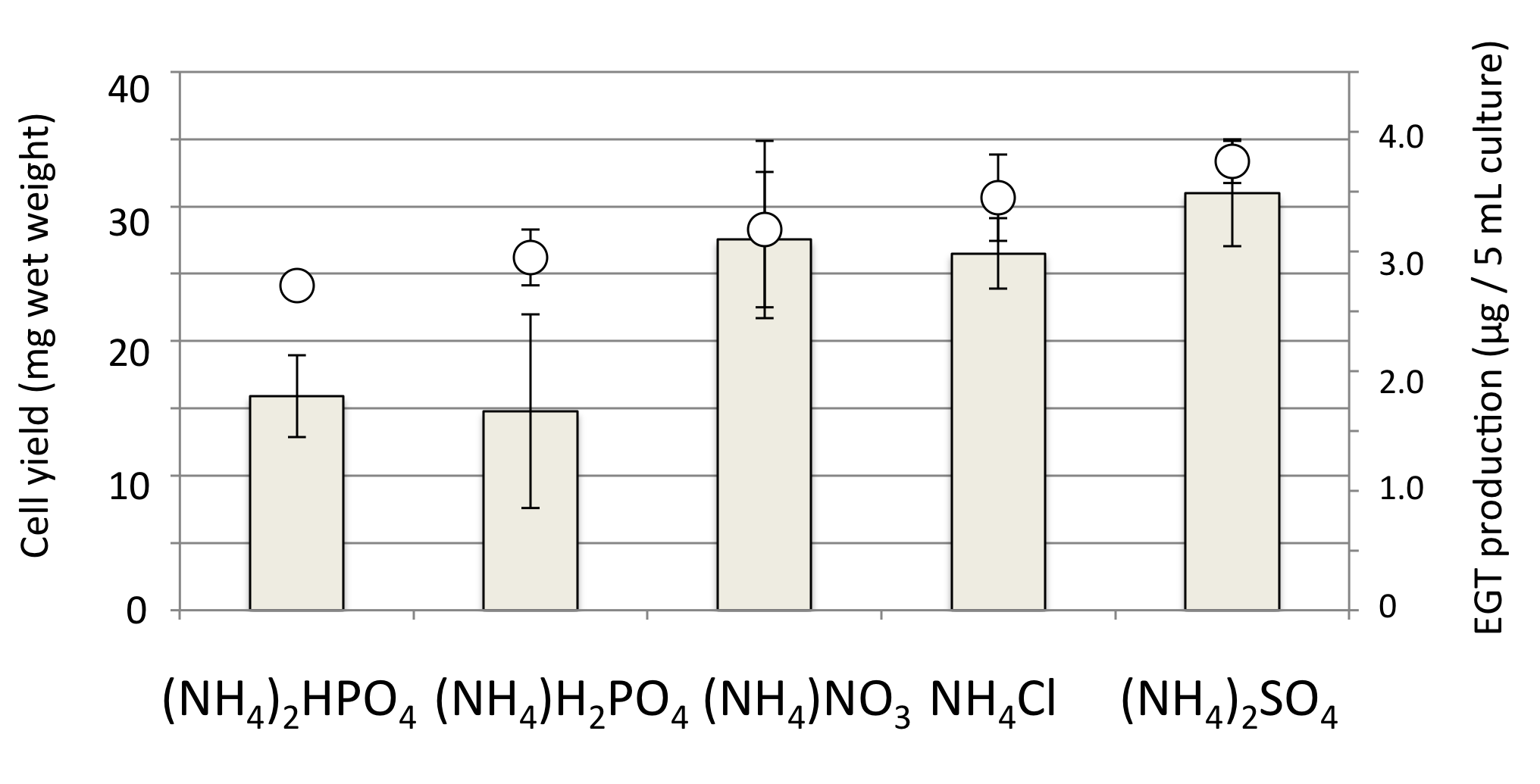


Figure S2. Effect of different nitrogen sources on EGT productivity of *Methylobacterium* sp. strain 22A grown on 0.5% methanol. Each nitrogen source was used at concentrations equivalent to 30 mM nitrogen. The experiment was done in five replicates using 5 ml media. The data are presented as the mean ± SD (n = 5). Bar, EGT production (µg/5 ml culture), and circles, cell yield (mg wet weight).


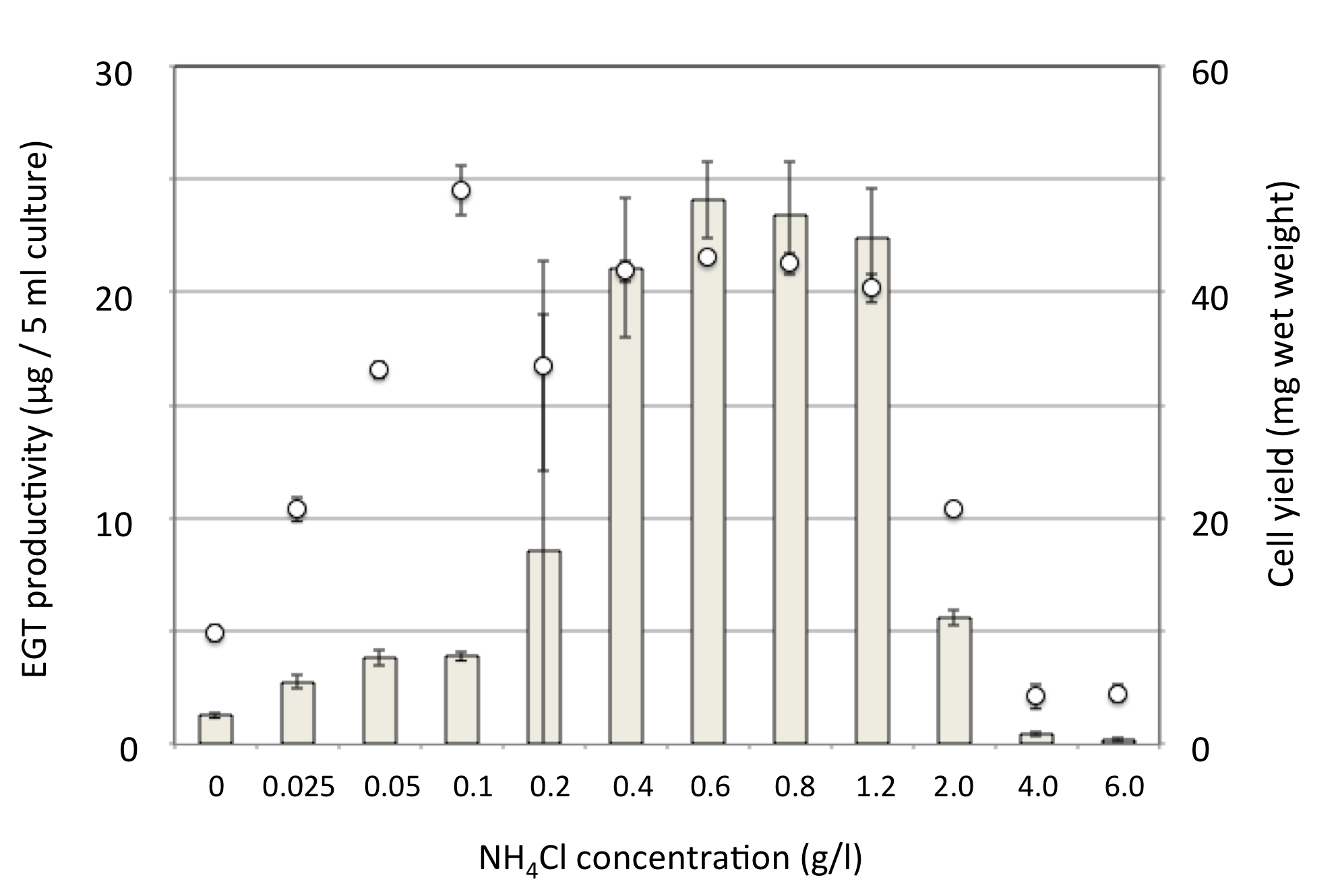


Figure S3. Effect of ammonium chloride concentration on EGT productivity of *Methylobacterium* sp. strain 22A grown on 0.5% methanol for 7 days. Original methanol medium contains 0.4 g/l NH_4_Cl. The experiment was done in triplicate using 5 ml media. The data are presented as the mean ± SD (n = 3). Bar, EGT production (µg/5 ml culture), and circles, cell yield (mg wet weight).


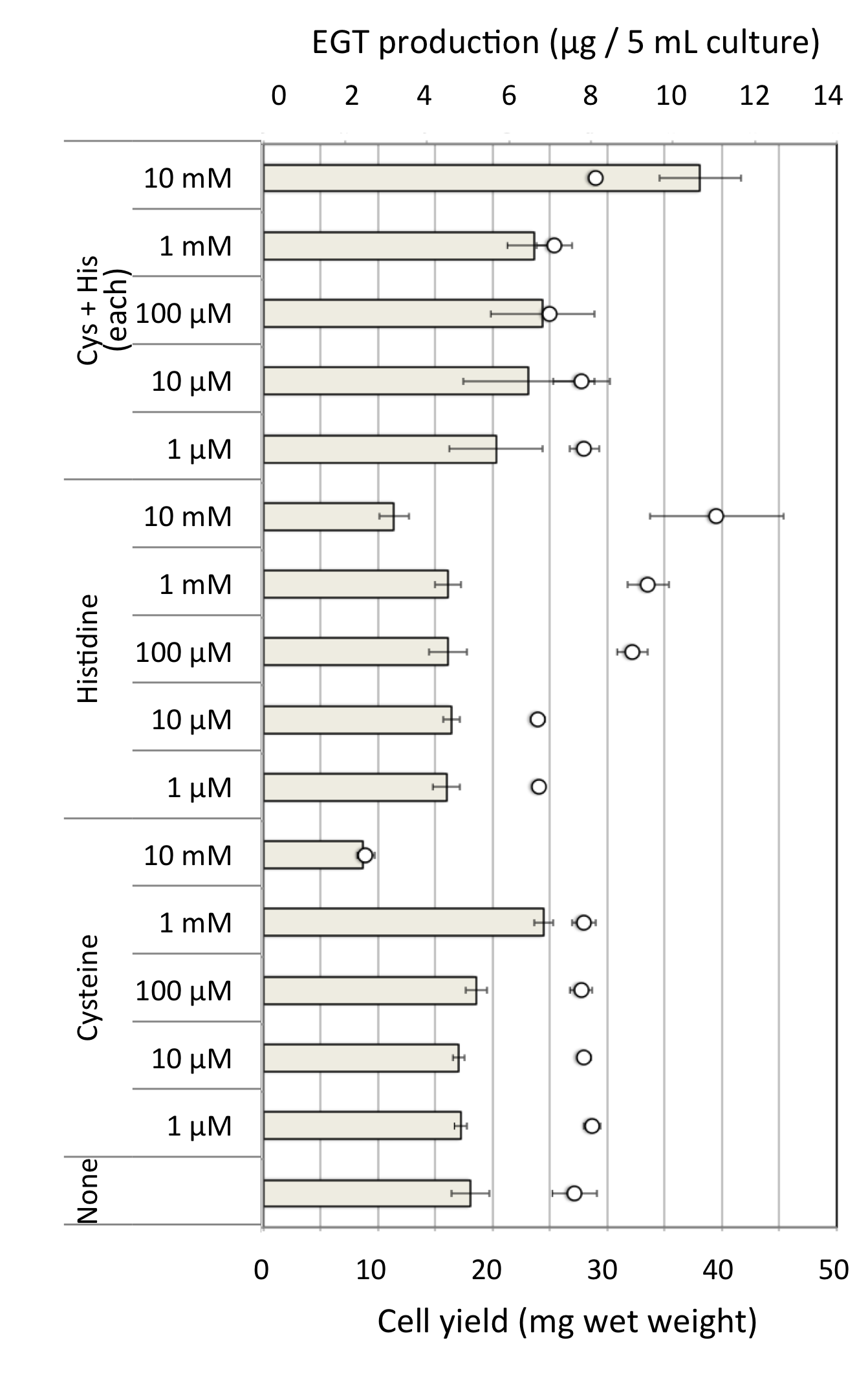


Figure S4. Effect of addition of histidine and cysteine on EGT productivity of *Methylobacterium* sp. strain 22A grown on 0.5% methanol for 7 days. Original methanol medium contains no amino acids (none). The experiment was done in triplicate using 5 ml media. The data are presented as the mean ± SD (n = 3). Bar, EGT production (µg/5 ml culture), and circles, cell yield (mg wet weight).


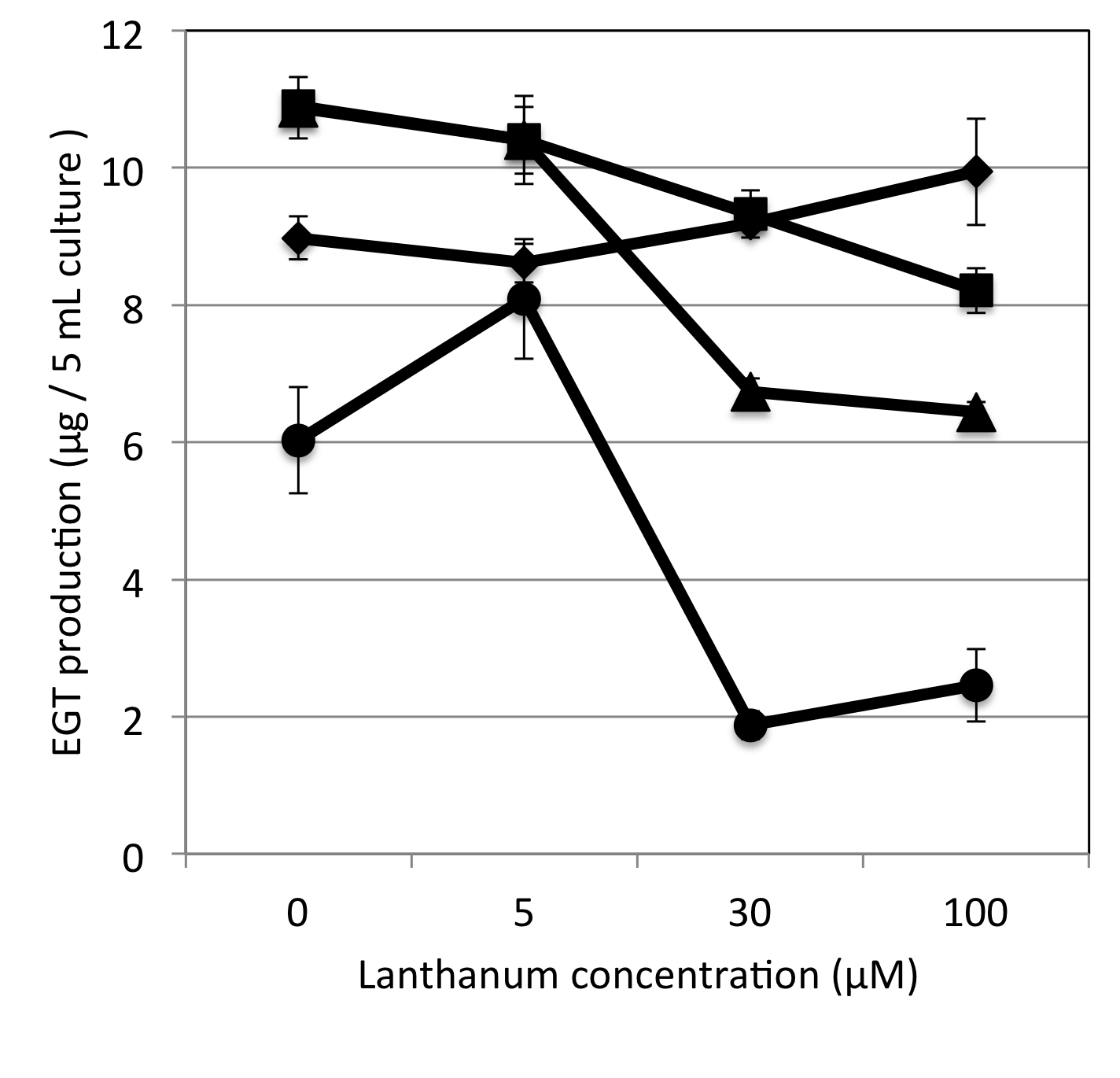


Figure S5. Effect of lanthanum addition on EGT productivity of *Methylobacterium* sp. strain 22A grown on various concentration of methanol for 7 days. Methanol concentration was 0.5% (circle), 1% (diamonds), 2% (squares), and 3% (triangles). The experiment was done in triplicate using 5 ml media. The data are presented as the mean ± SD (n = 3).


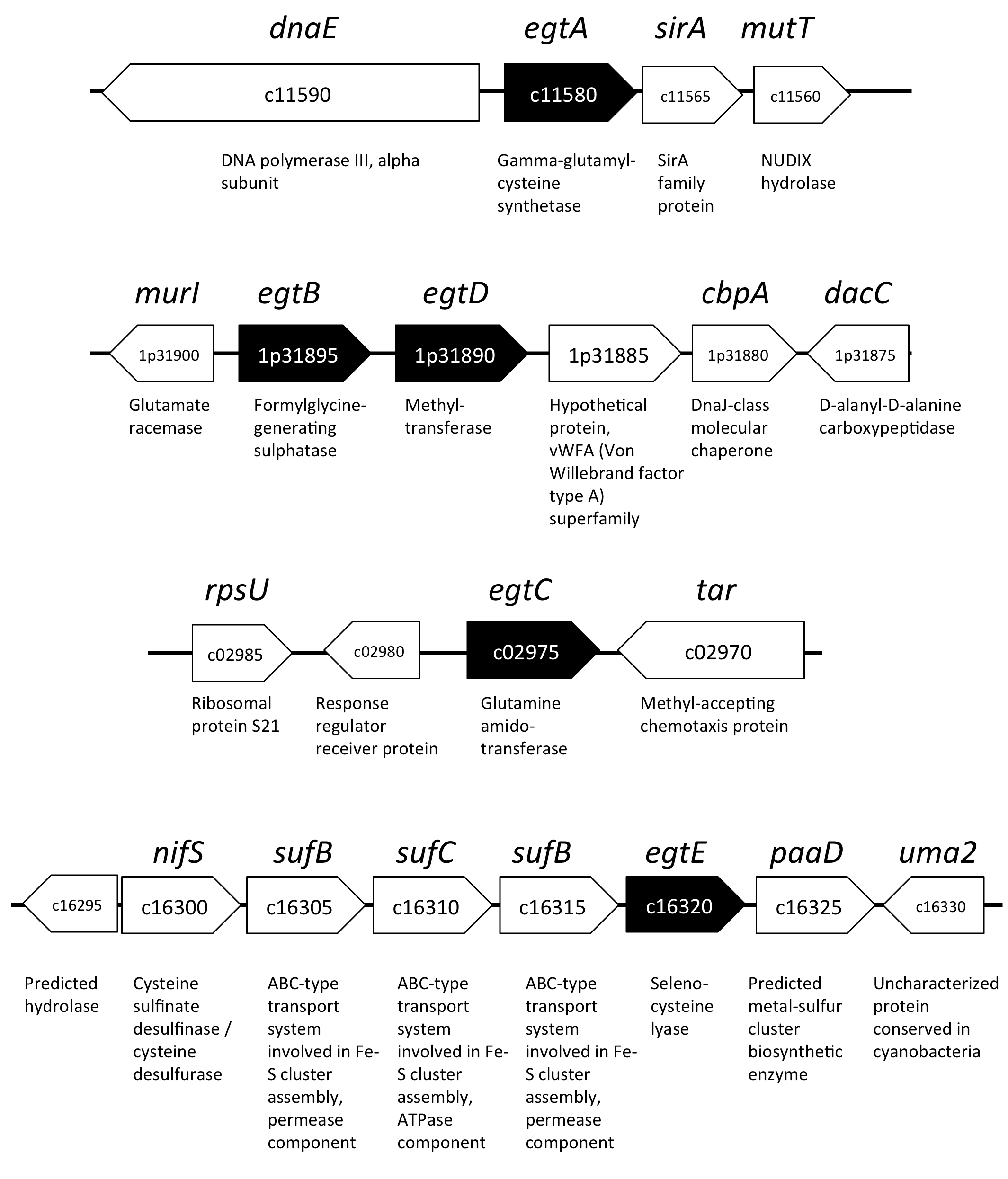


Figure S6. Gene organization of *egt* genes in the strain 22A genome. The gene sizes are shown in approximate size. *egt* genes are shown as filled arrows. Numbers in the arrows are locus tags in the strain 22A genome (Tani et al., 2015).


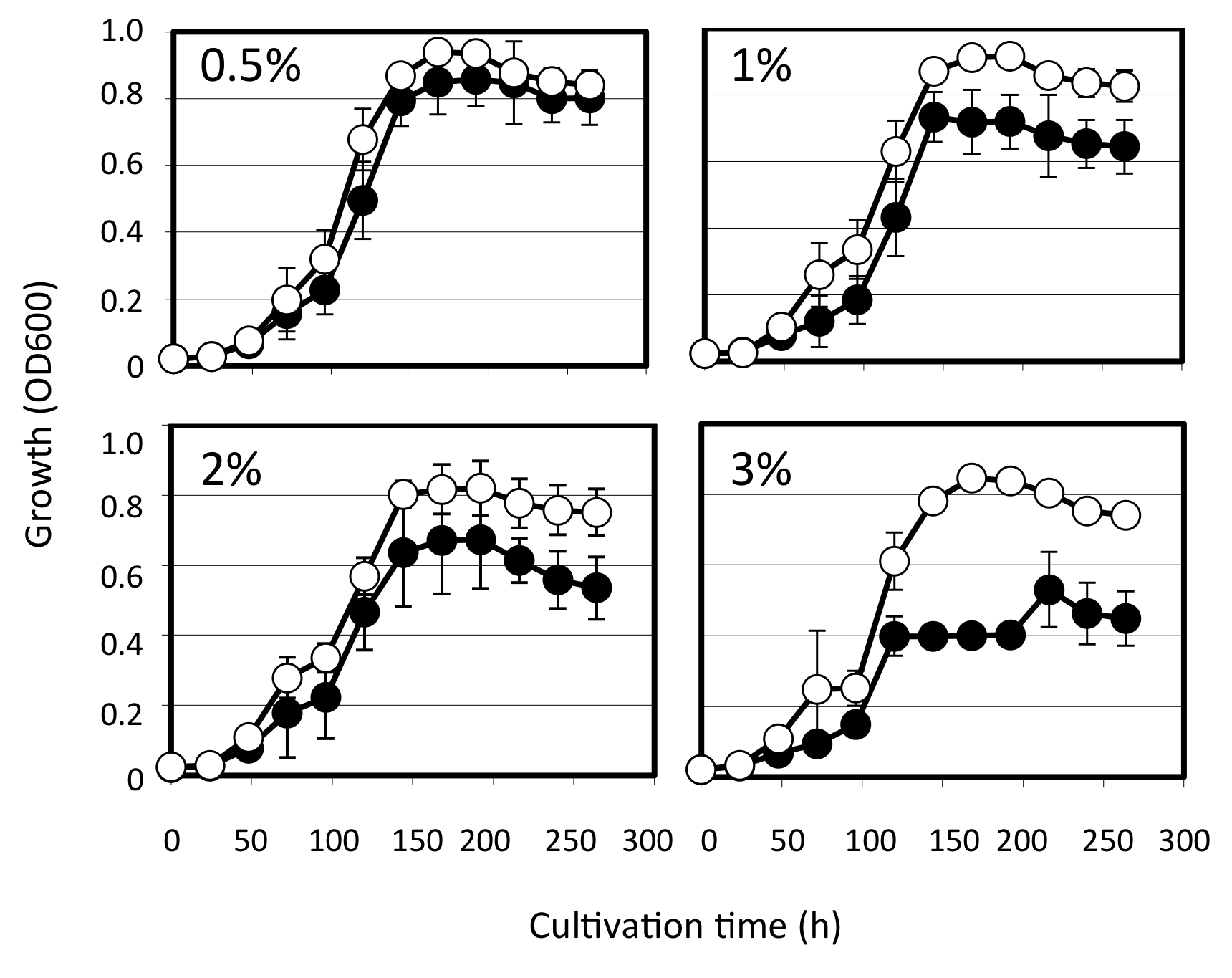


Figure S7. Growth of wild type and *Δegt* of strain 22A on different concentrations of methanol (0.5-3%). The cells were grown in 200 μl methanol media prepared in 96-well plates at 28°C. The cultivation was done in four technical replicates. Closed symbols, wild type; open symbols, *Δegt*. The data are presented as the mean ± SD (n = 4).


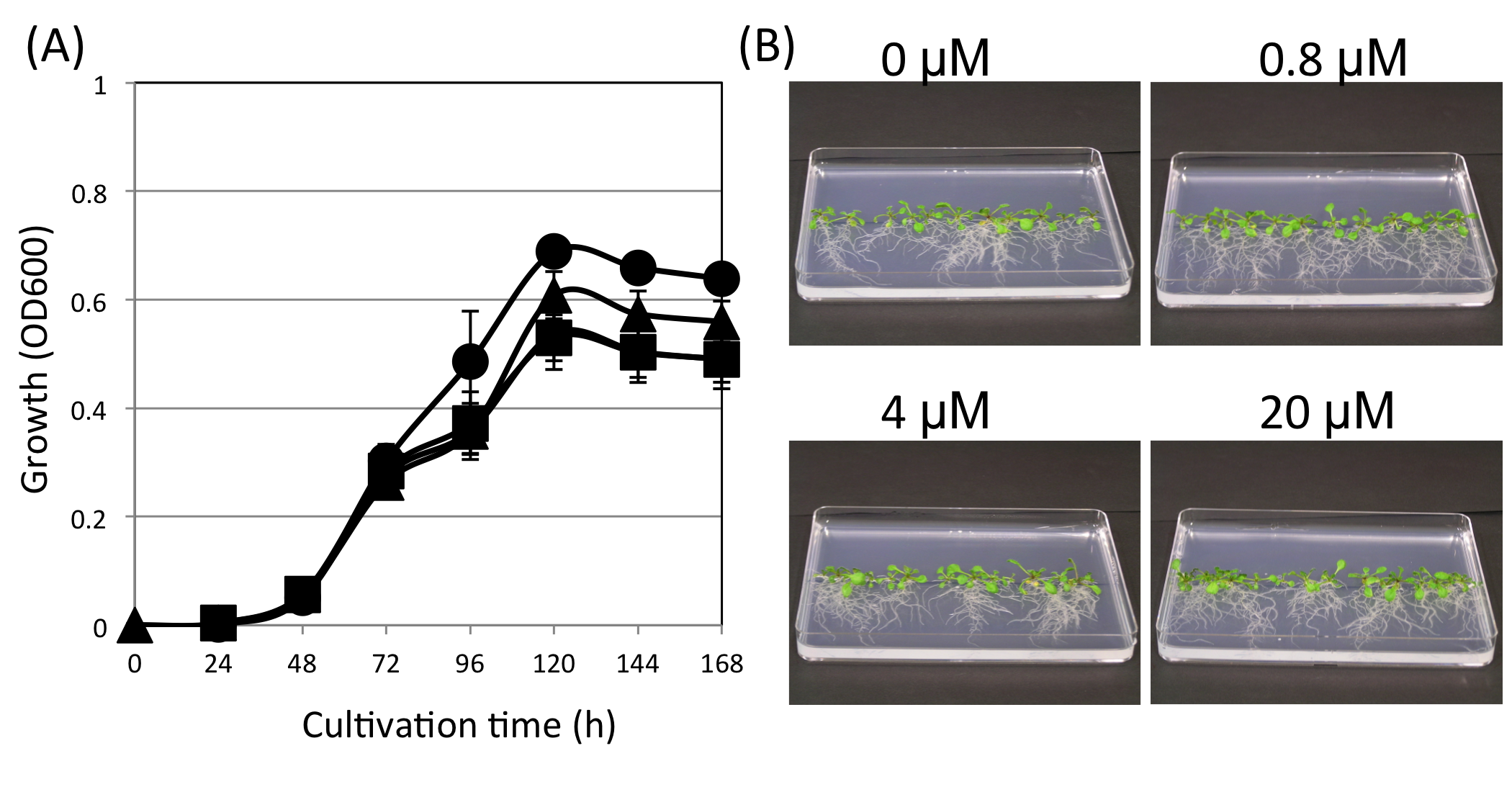


Figure S8. Growth of strain 22A wild type on methanol and *A. thaliana* in the presence of EGT. (A) Strain 22A was grown on methanol in 200 μl methanol medium in 96-well plates, supplemented with various concentrations of EGT. The data are presented as the mean ± SD (n = 4). Circle, 0 μM; triangles, 1 μM; squares, 10 μM; and diamonds, 100 μM EGT. (B) *A. thaliana* Col-0 grown on 1/2 MS agar in the presence of 0 to 20 μM EGT. Sterile seeds of *A. thaliana* Col-0 were placed on 1/2 MS agar medium, as described in Materials and Methods. The medium contained different concentrations of EGT (0, 0.8, 4, and 20 μM). The plates were incubated at 23°C under 16-/8-h light/dark conditions. The plates were tilted so that the roots could grow on the agar. After three weeks, the plant growth was observed.

Supplementary references

Tani A, Ogura Y, Hayashi T, Kimbara K: Complete genome sequence of *Methylobacterium aquaticum* strain 22A, isolated from *Racomitrium japonicum* moss. Genome Announc 3(2) e00266-15. (2015)
